# Efficacy and safety of dose-dense chemotherapy in urothelial carcinoma

**DOI:** 10.18632/oncotarget.16759

**Published:** 2017-03-31

**Authors:** Chenjing Zhu, Jiaming Liu, Jing Zhang, Qingfang Li, Qisi Lian, Jing Xu, Xuelei Ma

**Affiliations:** ^1^ Cancer Center, State Key Laboratory of Biotherapy, West China Hospital, Sichuan University, Chengdu, Sichuan, China; ^2^ Department of Urology, Institute of Urology, Laboratory of Reconstructive Urology, West China Hospital, Sichuan University, Chengdu, Sichuan, China; ^3^ West China School of Medicine, West China Hospital, Sichuan University, Chengdu, Sichuan, China; ^4^ West China School of Stomatology, Sichuan University, Chengdu, Sichuan, China

**Keywords:** dose-dense, urothelial carcinoma, meta-analysis

## Abstract

We conducted a meta-analysis to assess the efficacy and safety of dose-dense chemotherapy in the treatment of patients with urothelial carcinoma. A systematic search was conducted in PubMed, Medline, Embase, Web of Science and Cochrane Collaboration's Central register of controlled trials (CENTRAL) for relevant articles. Data was obtained from 10 trials with a total of 1093 patients. The pooled pathologic complete response (pCR) was 27.8% in the ten studies with a full cohort of 684 patients who received dose-dense methotrexate, vinblastine, adriamycin and cisplatin (dd-MVAC). In the controlled trials, although the difference was not significant, the pCR rate in the dd-MVAC group has a trend of increase (odds ratio (OR) 1.52; 95% confidence interval (CI) 0.78-2.98, *P =* 0.22) compared with classic MVAC group. A significant improvement of overall survival (OS) (hazard ratio (HR) 0.77, 95% CI 0.61–0.97, *p* = 0.03) was also observed. Hematologic toxicities were the most frequent grade ≥ 3 toxicities including neutropenia/febrile neutropenia (17.5%), anemia (9.4%) and thrombocytopenia (6.1%). Compared with the classic MVAC group, dd-MVAC was associated with significantly decreased risks of all-grade adverse events (AEs) such as anemia (OR 0.457, 95% CI 0.249–0.840, *p* = 0.012), febrile neutropenia (OR 0.398 95% CI 0.233–0.681, *p* = 0.001), and neutropenia (OR 0.373, 95% CI 0.201–0.691, *p* = 0.002). In conclusion, dose-dense chemotherapy was effective and tolerable in patients with urothelial carcinoma, which could be considered as a reasonable therapeutic option.

## INTRODUCTION

Urothelial carcinoma (UC) is a significant health problem. In 2008, it led to an estimated 150,200 deaths worldwide [1]. Twenty-five percent of UCs were invasive [2, 3]. According to the European Urology Association (EAU), radical cystectomy (RC) with bilateral pelvic lymphadenectomy was the gold standard treatment for patients with muscle-invasive tumors [4].

The classic MVAC regimen (methotrexate, vinblastine, doxorubicin and cisplatin) [5] was established as the most effective regimen (4-week-per-cycle) in the neoadjuvant chemotherapy setting [6], and the first recognized option for patients with locally advanced or metastatic UC [7]. In 1993, the classic MVAC regimen was modified into dose-dense MVAC (dd-MVAC) which was administered in cycles of 14 days. Dd-MVAC was shown to have fewer dose delays and a more favorable toxicity profile than classic MVAC group [8] in a randomized phase III trial, however in another study [9], similar pathological responses were observed between the two groups.

To systematically review the safety and efficacy of dose-dense chemotherapy, we assessed the pathologic complete response (pCR), objective response rate (ORR), overall survival (OS) and adverse events (AEs) of dose-dense chemotherapy, dd-MVAC in particular, in patients with UC. We also compared the efficacy and safety of dd-MVAC with classic MVAC.

## RESULTS

### Literature search

**Figure 1:**
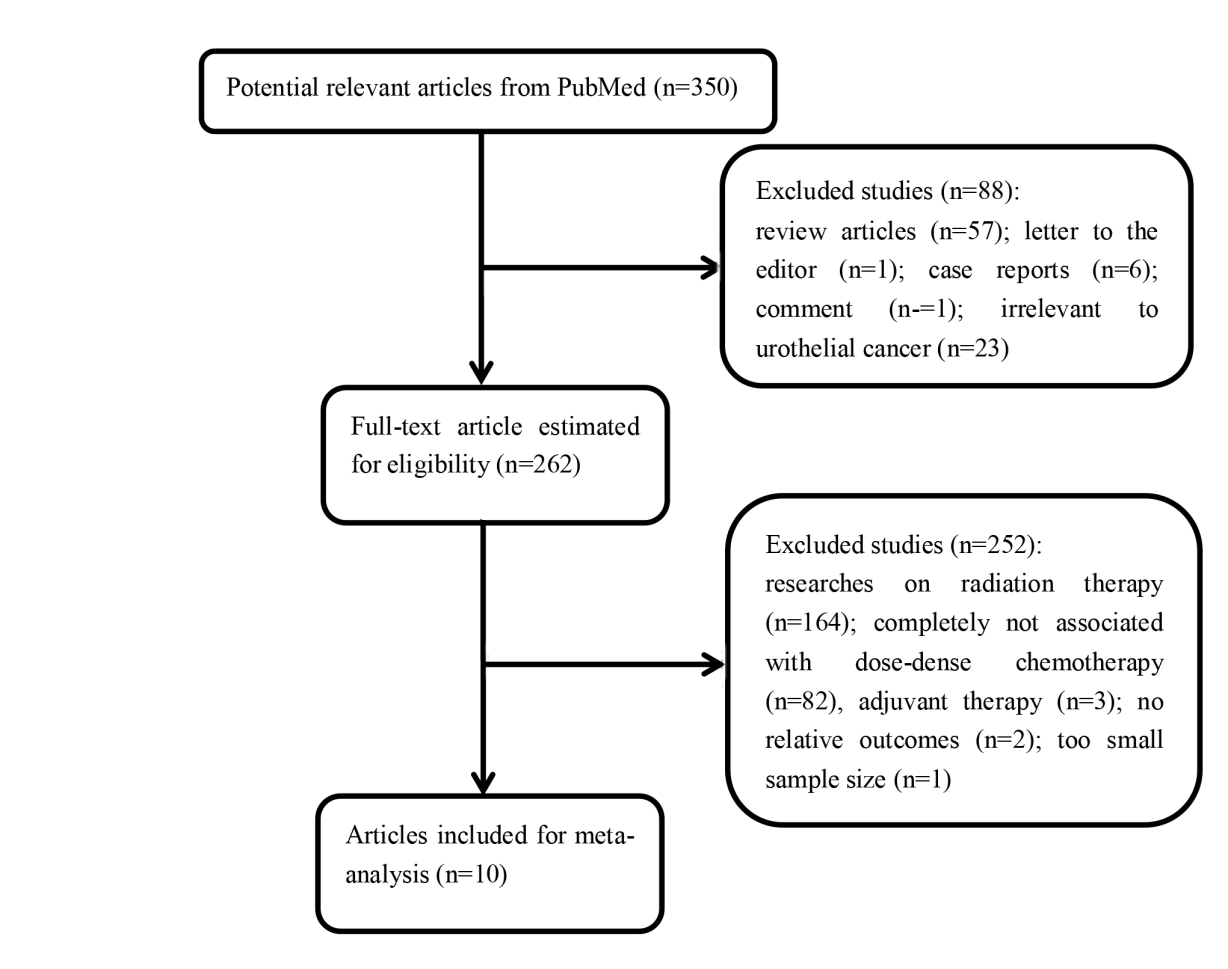
A flow diagram of the trial selection process

The initial search yielded 849 unique articles after deletion of duplicates. After title and abstract screening, 555 were excluded as they were case reports, letters, review articles, or irrelevant to urothelial cancer, leaving 294 articles for full review. After assessing the full texts of these potentially relevant studies, 284 were excluded for the following reasons: 189 were researches on radiation therapy, 85 were completely not associated with dose-dense chemotherapy, 5 were about adjuvant therapy, 4 contained no relative outcomes, and 1 was with a too small sample size. Ultimately, 10 eligible articles [7, 9–17] involving a total of 1093 patients were included for analyses. No additional unpublished trials were added to the literature search results. A flow diagram of the trial selection process is shown in Figure 1.

### Study characteristics

Results of the literature search identified 10 eligible papers. Among those papers, three [7, 9, 16] were controlled trials comparing dd-MVAC with classic MVAC, one [17] was another controlled trial comparing a dd-GC (gemcitabine 2500 mg/m2 and cisplatin 70 mg/m2, q 2 weeks) regimen with dd-MVAC which suggested that although not superior to dd-MVAC, dd-GC was better tolerated, and one [11] was AG-TC which was doxorubicin plus gemcitabine followed by paclitaxel plus carboplatin. The rest were single-arm trials exploring the safety and/or the activity of dd-MVAC regimen. Dd-MVAC chemotherapy consisted of methotrexate (MTX) 30 mg/m2, vinblastine (VBL) 3mg/m2, adriamycin (ADM) 30 mg/m2 and cisplatin (CDDP) 70 mg/m2, administered in cycles of 14 days. Classic MVAC was a 4-week-per-cycle regimen which was also the combination of MTX 30 mg/m2, VBL 3 mg/m2, ADM 30 mg/m2 and CDDP 70 mg/m2. The majority of the patients were men. The age ranged from 32 to 83 years. The basic characteristics of the included studies are detailed in Table 1.

**Table 1:** Basic characteristics of the included studies

Author	Year	Country	Tumor type	Type of study	No. of patients	Sex (Male/Female)	Median age (range) (y)	Tumor stage	Chemotherapy regimens (mg/m2)	No. of planned cycles	No. of pCR (total)
van de Putte EE	2016	Netherlands	Muscle invasive bladder cancer (MIBC)	Retrospective	dd-MVAC: 80	60/20	mean (SD) 57 (8)	T1-4 N0-3 M0-1	dd-MVAC (M 30, V 3, D 30, C 70 q 2 weeks)	4	23 (80)
					MVAC: 35	26/9	59 (9)		MVAC (M 30, V 3, D 30, C 70 q 4 weeks)	4	7 (35)
					GC: 51	36/15	63 (8)		GC (G 1000, C 70 q 3 weeks)	4	16 (51)
Bamias A	2013	Greek	Advanced urothelial cancer (UC)	Prospective, Phase III	dd-MVAC: 66	53/10	66 (35-76)	cT4b N2 N3	dd-MVAC (M 30, V 3, A 30, C 70 q 2 weeks)	≥ 6	5 (45)
					dd-GC: 64	55/8	65 (34-80)		dd-GC (G 2500, C 70 q 2 weeks)	≥ 6	5 (49)
Pouessel D	2016	France	Advanced urothelial bladder cancer	Retrospective	dd-MVAC: 189	160/29	63 (57-67)	T2-4a N1-3 M0	dd-MVAC (M 30, V 3, D 30, C 70 q 2 weeks)	4-6	58 (166)
					MVAC: 52	43/9	58 (55-66)		MVAC (M 30, V 3, D 30, C 70 q 4 weeks)	3-4	17 (45)
Sternberg CN	2005	Multicenter	Advanced urothelial cancer (UC)	phase III	dd-MVAC: 134	105/29	61 (36–76)	NA	dd-MVAC (M 30, V 3, D 30, C 70 q 2 weeks)	6	28 (134)
					MVAC: 129	107/22	62 (32–81)		MVAC (M 30, V 3, D 30, C 70 q 4 weeks)	4	12 (129)
Choueiri TK	2014	Multicenter	Muscle invasive urothelial cancer (MIUC)	Prospective, phase II	39	28/11	NA	cT2-cT4 N0-1 M0	dd-MVAC (M 30, V 3, D 30, C 70 q 2 weeks)	4	10 (36)
Galsky MD	2007	USA	Advanced Urothelial Carcinoma	Phase II	25	NA	67 (43–79)	NA	AG-TC*	5	5 (25)
Plimack ER	2014	Multicenter	Muscle invasive bladder cancer (MIBC)	Prospective, phase II	44	30/14	64 (44-83)	cT2-T4a N0-N1 M0	dd-MVAC (M 30, V 3, D 30, C 70)	3	15 (40)
Blick C	2012	UK	Muscle invasive bladder cancer (MIBC)	Retrospective	80	64/16	60 (41-74)	T2-4a N0-2 M0	dd-MVAC (M 30, V 3, D 30, C 70 q 2 weeks)	3 or 4	26 (60)
McConkey DJ	2015	USA	Muscle invasive urothelial cancer (MIUC)	Phase II	60	40/20	64 (42–79.6)	T1-4a N0 M0	dd-MVAC (M 30, V 3, D 30, C 70 q 2 weeks)	4	23 (60)
Edeline J	2012	France	Advanced bladder cancer	Retrospective	45	36/9	58 (36–79)	NA	dd-MVAC (M 30, V 3, D 30, C 70 q 2 weeks)	≥ 1	4 (38)

NA: not available.

AG–TC*: doxorubicin 50 mg/m2 plus gemcitabine 2000 mg/m2 every other week *5 cycles followed by paclitaxel 65 mg/m2 i.v. plus carboplatin 1.7 i.v. weekly *12 cycles.

### Response and survival

**Figure 2:**
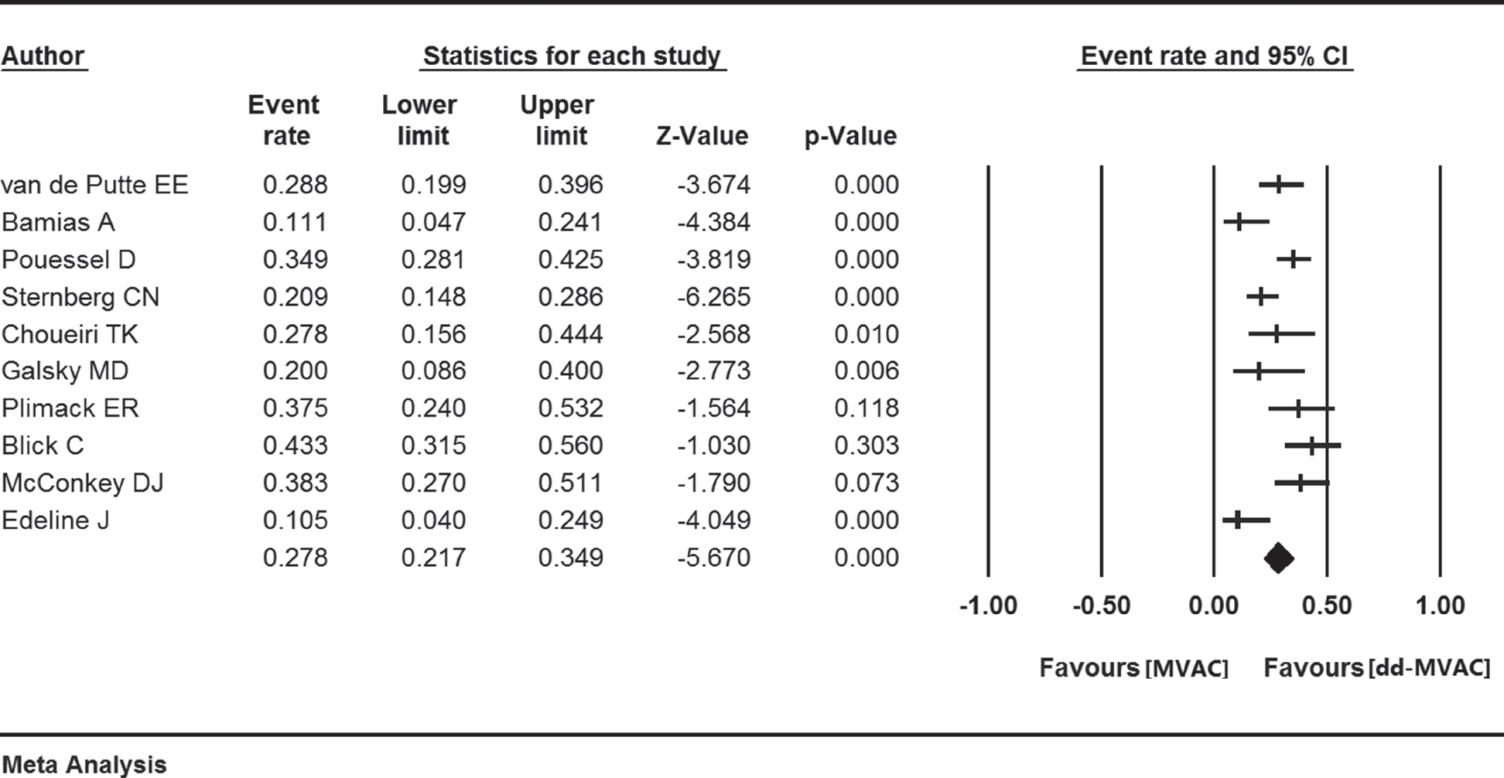
The pooled pathologic complete response (pCR) in the ten included studies

**Figure 3:**
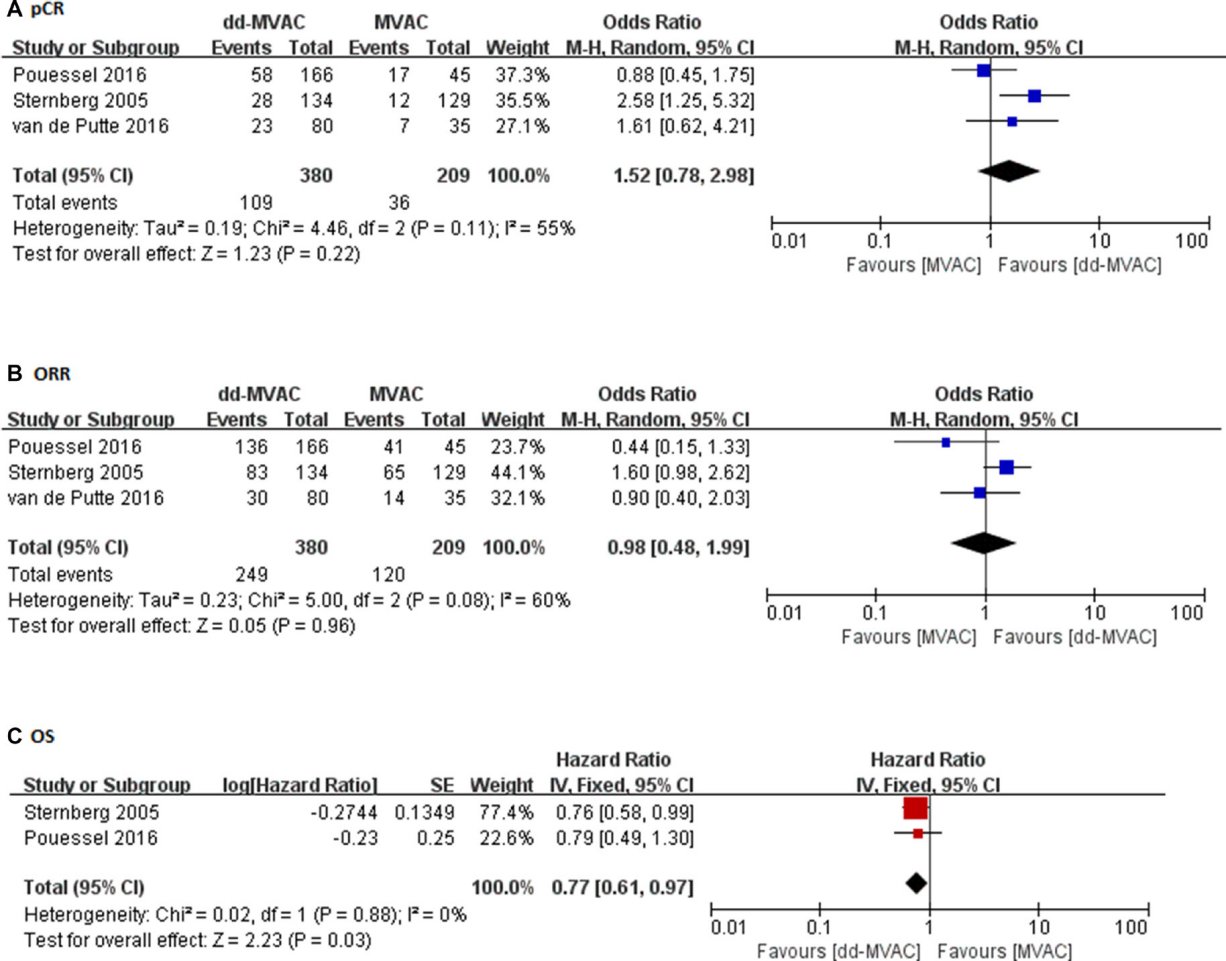
(**A**) The odds ratios of pathologic complete response (pCR) in the controlled trials comparing dd-MVAC and classic MVAC; (**B**) The odds ratios of objective response rate (ORR) in the controlled trials comparing dd-MVAC and classic MVAC; (**C**) Forest plots of the pooled hazard ratios (HRs) for OS.

In the ten studies with a full cohort of 684 patients who received dd-MVAC, the pooled pCR was 27.8% (95% confidence interval (CI) 0.217–0.349, *p <* 0.001) (Figure 2). The rate of pCR was presented in three controlled studies that compared dd-MVAC with classic MVAC. No significant difference was detected in the odds of achieving a pCR with dd-MVAC versus classic MVAC (odds ratio 1.52, 95% CI 0.78–2.98, *P =* 0.22, *I*^2^ = 55%, random-effects model) (Figure 3A). In addition, there was no significant improvement in terms of ORR between the dd-MVAC group compared with classic MVAC group, with an odds ratio of 0.98 (95% CI 0.48–1.99, *p =* 0.96, I^2^ = 60%, random-effects model) (Figure 3B).

As for long-term survival, a significantly improved OS was noted with dd-MVAC treatment when compared with classic MVAC treatment (hazard ratios (HR) 0.77, 95% CI 0.61–0.97, *p =* 0.03, *I*^2^ = 0%, fixed-effects model) (Figure 3C).

### Toxicity

**Figure 4:**
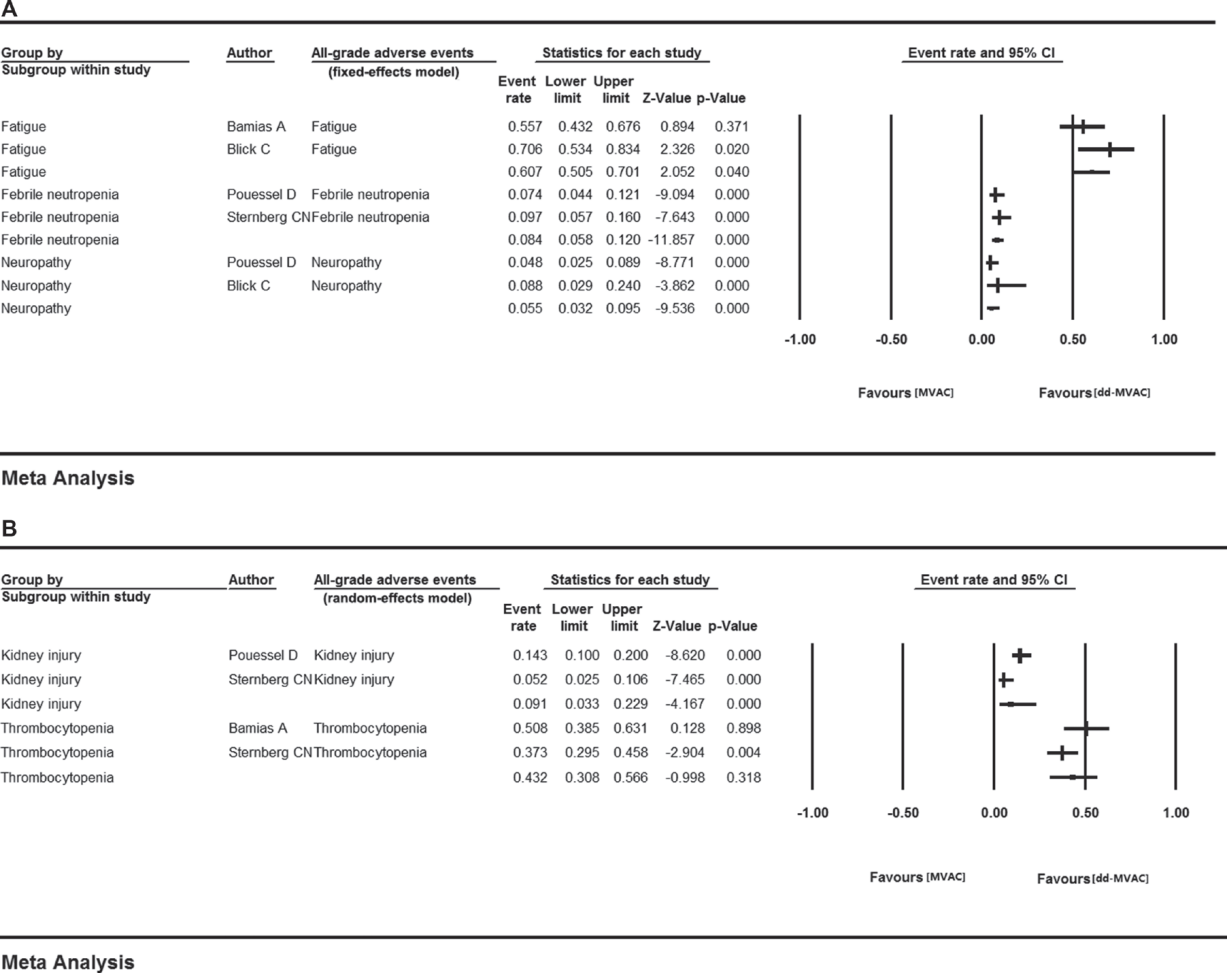
All-grade AEs in the ten included studies (**A**) fixed-effects model; (**B**) random-effects model.

We analyzed the rates of all-grade and grade 3 or more AEs in the ten included studies to evaluate the safety of dose-dense chemotherapy. In all-grade AEs, the highest risk was found for fatigue (60.7%, 95% CI 0.505–0.701, *I*^2^ = 49.967%, fixed-effects model). Thrombocytopenia was also common with the event rate of 43.2% (95% CI 0.308–0.566, *I*^2^ = 67.955%) using a random-effects model (Table 2, Figure 4).

**Table 2:** The rate of all-grade and grade ≥ 3 adverse events (AEs) for dose-dense MVAC

All-grade adverse events	Model	Event rate with 95% CI	I^2^
Fatigue	Fixed model	0.607 (0.505–0.701)	49.967
Febrile neutropenia Neuropathy	Fixed model Fixed model	0.084 (0.058–0.120) 0.055 (0.032–0.095)	0.000 0.000
Kidney injury Thrombocytopenia	Random model Random model	0.091 (0.033–0.229) 0.432 (0.308–0.566)	84.160 67.955

Grade ≥ 3 adverse events	Model	Event rate with 95% CI	I^2^
Dehydration Fatigue Hyponatremia Kidney injury Mucositis Nausea/vomiting Neuropathy Sepsis	Fixed model Fixed model Fixed model Fixed model Fixed model Fixed model Fixed model Fixed model	0.051 (0.019–0.128) 0.077 (0.049–0.118) 0.019 (0.005–0.074) 0.025 (0.012–0.052) 0.029 (0.009–0.087) 0.046 (0.022–0.094) 0.011 (0.002–0.052) 0.074 (0.038–0.142)	0.000 4.430 0.000 9.112 0.000 0.000 0.000 42.331
Stomatitis Thrombosis Anemia Neutropenia/febrile neutropenia Thrombocytopenia	Fixed model Fixed model Random model Random model Random model	0.038 (0.012–0.112) 0.038 (0.016–0.088) 0.094 (0.035–0.228) 0.175 (0.096–0.296) 0.061 (0.020–0.175)	11.398 0.000 87.575 84.806 84.951

**Figure 5:**
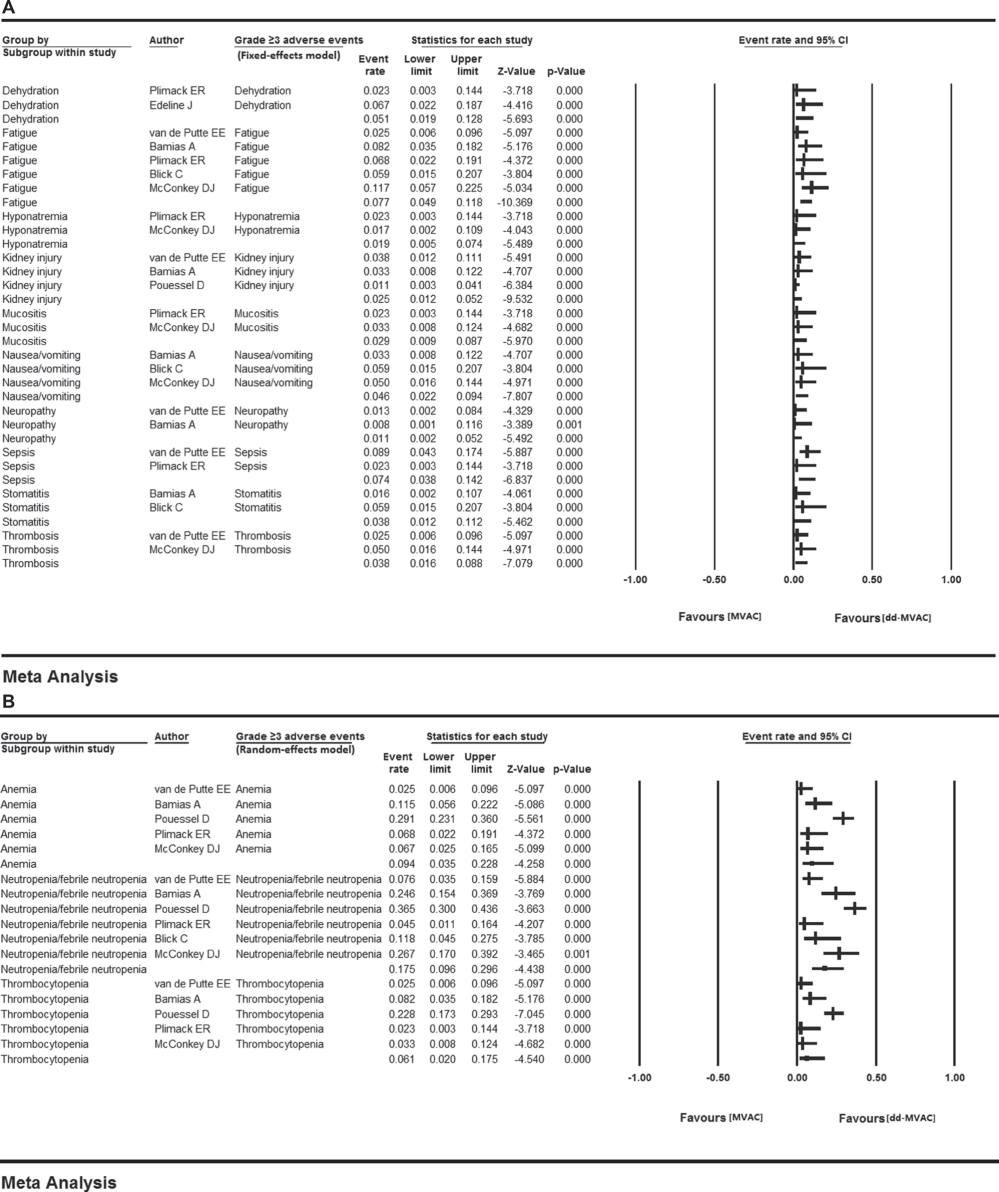
Grade 3 or more AEs in the ten included studies (**A**) fixed-effects model; (**B**) random-effects model..

The grade ≥ 3 toxicities were predominantly hematologic. Neutropenia/febrile neutropenia (17.5%, 95% CI 0.096–0.296, *I*^2^ = 84.806%, random-effects model), anemia (9.4%, 95% CI 0.035–0.228, *I*^2^ = 87.575%, random-effects model), fatigue (7.7%, 95% CI 0.049–0.118, *I*^2^ = 4.430%, fixed-effects model), sepsis (7.4%, 95% CI 0.038–0.142, *I*^2^ = 42.331%, fixed-effects model), and thrombocytopenia (6.1%, 95% CI 0.020–0.175, *I*^2^ = 84.951%, random-effects model) were the most frequent high-grade treatment-related adverse events (Table 2, Figure 5).

**Figure 6:**
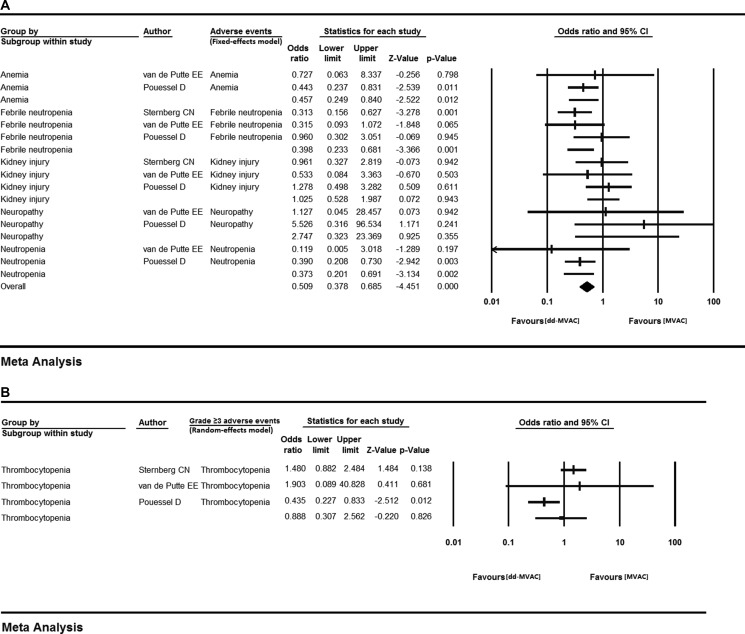
The odds ratios of adverse events (AEs) in the controlled trials comparing dd-MVAC and classic MVAC (**A**) fixed-effects model; (**B**) random-effects model.

We further calculated the OR of all-grade adverse events between the dose-dense chemotherapy and classic chemotherapy groups. No statistical heterogeneity was observed in each adverse event except for thrombocytopenia, which was analyzed with a random-effects model. The results showed that compared with the classic MVAC group, dd-MVAC was associated with decreased risks of anemia (OR 0.457, 95% CI 0.249–0.840, *p =* 0.012, I^2^ = 0.000, fixed-effects model), febrile neutropenia (OR 0.398 95% CI 0.233–0.681, *p =* 0.001, I^2^ = 29.370%, fixed-effects model), and neutropenia (OR 0.373, 95% CI 0.201–0.691, *p =* 0.002, I^2^ = 0.000, fixed-effects model). Increased risks of kidney injury (OR 1.025, 95% CI 0.528–1.987, *p =* 0.943, I^2^ = 0.000, fixed-effects model) and neuropathy (OR 2.747, 95% CI 0.323–23.369, *p =* 0.355, I^2^=0.000, fixed-effects model) (Table 3, Figure 6) were found for dd-MVAC, but there were no significant differences.

**Table 3:** All-grade adverse events of dd-MVAC versus MVAC

All-grade adverse events	Odds Ratio with 95% CI	Model	I^2^
Anemia	0.457 (0.249–0.840)	Fixed model	0.000
Febrile neutropenia	0.398 (0.233–0.681)	Fixed model	29.370
Kidney injury	1.025 (0.528–1.987)	Fixed model	0.000
Neuropathy	2.747 (0.323–23.369)	Fixed model	0.000
Neutropenia	0.373 (0.201–0.691)	Fixed model	0.000
Thrombocytopenia	0.888 (0.307–2.562)	Random model	76.634

### Risk of bias and quality assessment

**Figure 7:**
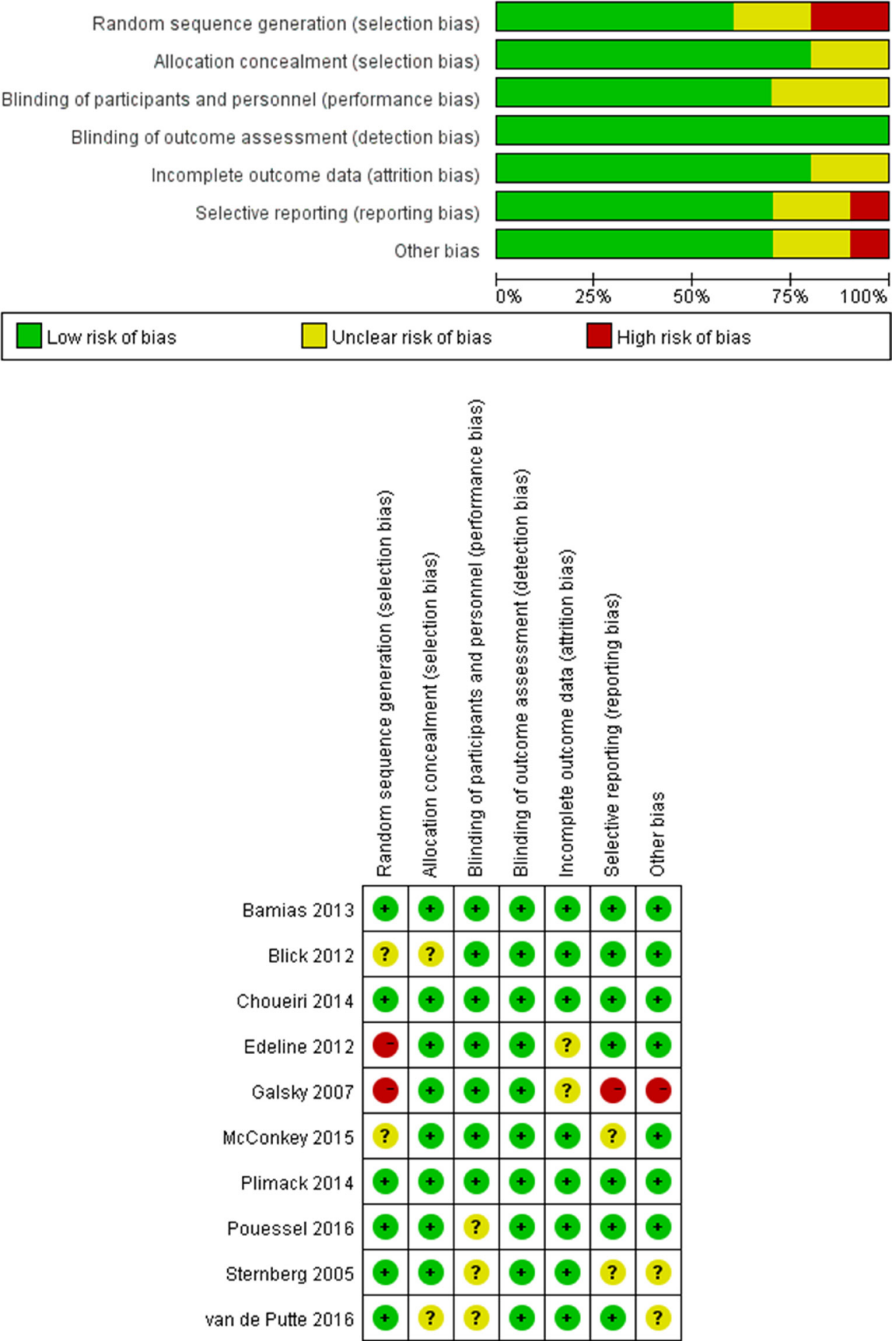
Risk of bias and quality assessment (**A**) Risk of bias graph: review authors’ judgments about each risk of bias item presented as percentages across all included studies; (**B**) Risk of bias summary: review authors’ judgments about each risk of bias item for each included study.

The risk of bias and quality assessments of the included studies are outlined in Figure 7A, 7B. The Jadad score of two controlled trials [7, 16] were 3 and one [9] got a score of 2. Overall, the quality of the included studies was satisfactory.

## DISCUSSION

To the best of our knowledge, this is the first meta-analysis that assesses the tolerability and activity of dose-dense chemotherapy in patients with urothelial carcinoma. Dose-dense MVAC led to a significant increase in long-term survival and a decrease in all-grade adverse events. The pCR rate in the dose-dense group had a trend of increase compared with the classic chemotherapy group, although the difference was not statistically significant.

The degree of pathologic response is correlated with survival after UC surgery [18]. A meta-analysis demonstrated that patients with UC who achieved pCR after neoadjuvant chemotherapy had better OS than do patients without pCR. In breast cancer settings, the relationship between pCR and OS has been obscure, sometimes significantly associated [19, 20], but sometimes not [21, 22]. In our study, we assumed that the pCR rate and OS tended to increase in the dose-dense group, and no statistical difference might largely rely on the limited sample size.

The improved overall survival represented the advantage of long-term survival in dd-MVAC group, which was presumed to arise from the elimination of micrometastatic disease, instead of from improved local disease control [13]. In addition, better median progression-free survival was suggested by one study [16] with dd-MVAC (9.5 months) vs. classic MVAC (8.1 months) (HR = 0.73, 95% CI 0.56–0.95, *P =* 0.017).

Concomitantly, the toxicity data demonstrated that dd-MVAC was associated with a better tolerability than classic MVAC. The fewer adverse events were most probably due to bone marrow support with the routine addition of G-CSF [7, 16]. Toxicities may cause a delay in cystectomy or even deaths. All-grade AEs such as fatigue and thrombocytopenia were the most common, and it is suggested that surgeons still need to pay particular attention to grade ≥ 3 hematologic toxicities including neutrophil, erythrocyte and platelet toxicities.

The introduction of dose-dense combination chemotherapy provides advantages over standard chemotherapy. First, dose-dense chemotherapy has the potential to increase response rates as well as reduce the risk of progression and death [23, 24]. Second, a large proportion of patients achieved the planned number of cycles without serious life-threatening toxicity with the administration of G-CSF.

What’s more, it is important to identify the optimal cycle of dd-MVAC. The number of cycles was variable in the included publications, but most studies adopted 3–5 cycles which displayed less toxicity and fewer dose delays.

There are several limitations in our study. First, because this dose-dense chemotherapy regimen is brought forward only recently which is rather new, there are few clinical trials about it. Second, the heterogeneity in our analysis could arise from the heterogeneous study population with pre-treated disease and the small sample size. Nevertheless, we used the random-effects model to reduce heterogeneity and also performed risk of bias and quality assessments and the Jadad score analyses to assess the quality of the included studies which turned out to be satisfactory.

Our systematic analyses of prospective and retrospective experiences with dd-MVAC suggested similar efficacy combined with better tolerability. We consider that the use of dose-dense chemotherapy regimen could be a trend in future treatment of patients with urothelial carcinoma. Further randomized trials comparing the two regimens are needed.

## MATERIALS AND METHODS

### Literature search

We carried out a systematic literature search up to Nov 21, 2016 in PubMed, Medline, Embase, Web of Science and Cochrane Collaboration’s Central register of controlled trials (CENTRAL), using the following search keywords: (dose dense OR dose-dense OR high-dose intensity OR accelerated OR dose-intensified OR dose-intensive) and (urothelial OR bladder) and (cancer OR carcinoma). There was no limit on the language of publications. We also carried out further searches for relevant unpublished trials in the clinical trial registry (http://www.clinicaltrials.gov).

### Study selection criteria

The inclusion criteria for the current analysis were that studies (i) included patients diagnosed with urothelial cancer; (ii) evaluated dose-dense chemotherapy; (iii) reported survival outcomes of patients (pCR or OS or PFS or ORR); and (iiii) evaluated the toxicities.

Studies were excluded if (i) they were review articles, letters, comments, or case reports; (ii) they evaluated radiotherapy, or high-dose rather than dose-dense chemotherapy; (iii) the sample size was less than 20 patients. The study selection process was performed according to PRISMA flow diagrams. Two reviewers selected studies independently. Any disagreements were resolved through discussion with another author.

### Data extraction

We extracted the first author, year of publication, tumor type, study phase, sample size, chemotherapy regimens, the number of cycles of chemotherapy, patient characteristics (number of patients, sex, median age, and tumor stage). The number of patients achieving pCR and ORR, HRs for OS, and the number of chemotherapy-induced adverse events were also extracted from the papers.

### Statistical analysis

The primary clinical endpoint used for the study was pCR, defined as the absence of histological evidence of invasive tumor cells (pT0N0M0 stage) [25]. The secondary clinical endpoints included (i) ORR, which was the summation of partial and complete response rates [26], (ii) OS, defined as the time from diagnosis of primary tumor to death, which can be from any cause [27], or the time to last contact; (iii) adverse events, which were graded according to the National Cancer Institute (Washington DC, USA) Common Toxicity Criteria.

For dichotomous outcomes, we calculated the odds ratio (OR) and 95% confidence intervals (CIs). For time-to-event data, we pooled the HR to compare the risk of death between the treatment group and the control group, using the generic inverse variance facility of RevMan [28]. A 2-tailed *p* value of less than 0.05 was considered as statistically significant. Statistical heterogeneity was defined as I^2^ > 50% and *P* ≤ 0.1. If the heterogeneity existed, we used the random-effects model. All analyses were done with Comprehensive Meta-Analysis (CMA) program 2 (Biostat, Englewood, NJ) and Review manager 5.3 (Copenhagen, Sweden).

### Risk of bias and quality assessment

The assessment of risk of bias and the quality of the included studies was measured using Review Manager 5.3 (Copenhagen, Sweden). We applied the assessment tool QUADAS-2, which consisted of four key domains: patient selection, index test, reference standard and flow and timing. Risk of bias was rated as high/low/unclear. Two authors independently applied the risk of bias tool and differences were resolved by discussion with a third author.

We also utilized the Jadad scale to assess the qualities of the randomized controlled trials included in our study [29]. A score lower than 2 indicated that the clinical trial was of low quality while a score more than 3 indicated a high-quality design [30]. Two authors independently calculated the scores and differences were resolved by discussion with a third author.
